# High genetic diagnostic yield in children and adolescents with psychiatric disorders

**DOI:** 10.1192/j.eurpsy.2023.292

**Published:** 2023-07-19

**Authors:** C. Manso-Bazús, N. Spataro, L. Torrent, L. Plans, M. Casadesús, M. Tomás, N. Baena, J. P. Trujillo, N. Capdevila, A. Brunet, V. Martínez-Glez, M. Pàmias, A. Ruiz Nel·lo

**Affiliations:** 1 Center of Genomic Medicine; 2 Genetics Laboratory. Center of Genomic Medicine; 3 Children and adolescescents Mental Health Service, Hospital Parc Taulí, Sabadell; 4 Servicio SESM-DI Catalunya central., Fundación Althaia, Manresa, Spain

## Abstract

**Introduction:**

Psychiatric disorders are more prevalent in children with mild (MID) to borderline intellectual functioning (BIF). Rare pathogenic variants in neurodevelopmental genes increase the risk for psychiatric disorders and may explain the comorbidity. Despite these patients represent up to 35% of those attended at mental health services, genetic diagnosis is usually not offered. The identification of mentioned variants could lead to improved clinical care.

**Objectives:**

To identify pathogenic variants responsible of the psychiatric disorders in mild and borderline intellectual functioning.

To correlate phenotypic and genetic profiles to personalize diagnostic, clinical care and support to clinicians and families.

**Methods:**

Whole exome sequencing (WES) was performed on 99 enrolled children/adolescent (6-18 yo) affected by a psychiatric condition diagnosed following DSM-5 criteria, and either MID (IQ 55-69) or BIF (IQ 70-85). Severity and interference of IQ and psychiatric comorbidity was evaluated using several psychometric tests (Conners, CDI, STAIC, CAARMS, CBCL and hONOSCA). Inheritance pattern was assessed through Sanger sequencing. ACMG/AMP guidelines were used for variant classification.

**Results:**

In our cohort, 64% patients presented BIF and 36% MID. 45% of the patients had 2 or more psychiatric diagnoses, the most prevalent (87%) being attention deficit hyperactivity disorder and, in second place, autism spectrum disorder (51%).

WES identified pathogenic/likely pathogenic variants in 30% of analyzed patients (30/99), 80% of the variants were de novo. There is no significant difference in patient severity between those with a genetic diagnosis and those without.

**Conclusions:**

Rare deleterious and *de novo* variants in neurodevelopmental genes are responsible for the comorbidity that exists between psychiatric disorders and mild/borderline intellectual disability.

The high diagnostic yield obtained from our exome sequencing approach demonstrates the need to offer genetic testing in children with psychiatric disorders and comorbid mild to borderline intellectual functioning.

Finally, patients being identified with a genetic diagnosis are subsequently attended in a specialised unit for rare disorders to receive personalised clinical management.

**Disclosure of Interest:**

None Declared

